# How does general and specific human capital drive technology development in firms?

**DOI:** 10.1371/journal.pone.0337056

**Published:** 2025-11-20

**Authors:** Jihoon Shin, Miguel Amaral, Ana Cristina Barros, M. Granger Morgan

**Affiliations:** 1 Innovation, Policy, and Entrepreneurship Thrust, Hong Kong University of Science and Technology (Guangzhou), Guangzhou, Guangdong, China; 2 Department of Engineering and Management, Instituto Superior Técnico, Lisbon, Portugal; 3 INESC TEC, Campus da FEUP, Porto, Portugal; 4 Department of Engineering and Public Policy, Carnegie Mellon University, Pittsburgh, Pennsylvania, United States of America; Universidade Federal do Tocantins, BRAZIL

## Abstract

This paper examines the extent to which human capital within a firm is associated with technology development. We develop and apply an original and more objective measure of technological advancement based on companies’ transitions from their current economic activity into a new one involving a higher or lower level of technological intensity. We use data from 2009 to 2015, when CAE Rev.3 was adopted, to ensure better harmonization of the dataset. The study focuses on technology intensity within manufacturing industries, based on two-digit CAE codes. We use an extremely rich longitudinal matched employer-employee micro dataset, Quadros de Pessoal, which covers nearly all Portuguese private companies and their employees. Our results show that three factors contribute positively and significantly to technology development: education level, prior work experience in the same industry, and the combination of STEM education with prior work experience in the same industry. This paper advances understanding of the link between human capital and technology development by introducing an objective measure of technological advancement, adopting a conceptual framework that emphasizes the combined effects of human capital, and demonstrating the value of matched employer-employee microdata for analyzing firms’ technological trajectories. The findings also provide managerial and policy insights into how to increase the likelihood and speed of technology development.

## Introduction

Firms must continue to innovating in order to retain their position in global markets [1]. Accordingly, both governments and firms consider technology to be a critical factor in improving firm performance and accelerating industrial development [2–6]. The literature has focused on a variety of roles that human capital plays in shaping and influencing technological development. A number of authors find that mobility of engineers and scientists facilitates technology transfer and enables firms to absorb foreign technologies [7–9]. Higher education has long been discussed as a precondition for technological development in the sense that education is the key process of facilitating learning and the acquisition of knowledge [10–12]. Much of the literature analyses the influence of the education and experience of firm founders and top management teams on technology development. Kato et al. [13] highlight that a highly educated and experienced founder is more likely to encourage R&D activities and boost innovative outcomes. Alexieve et al. [14] and Talke et al. [15] show that diversified educational background and experience of managers provide heterogeneous perspectives that are beneficial to innovation.

Although scholars broadly agree that human capital is essential for technology development [16–24], there is little agreement on which dimensions of human capital most directly influence technological progress. Human capital can be defined in terms of education, professional experience, skills, and organizational practices, but it is not easy to determine which of these dimensions has the greatest impact or how their effects vary across contexts. This multifaceted nature makes it difficult to evaluate the extent to which specific forms of human capital contribute to technology development.

The debate is particularly evident in research on education and its relationship with technology development. For example, McGuirk et al. [20] found that higher levels of education had no significant effect on product, service, or process development in Irish firms, regardless of firm size. In contrast, Capozza and Divella [25] showed that tertiary education in emerging economies was positively associated with new product development, though unrelated to process development or a firm’s capacity to generate technology internally. These mixed findings illustrate that while education is central to the concept of human capital, its precise contribution to technology development remains contested.

Professional working experience adds further complexity. De Winne and Sels [26] reported that managers’ industry-specific experience could negatively affect technological outcomes, as adherence to established routines may stifle experimentation and adaptation. Similarly, Guo et al. [16] found that Chinese top managers with military backgrounds invested less in technological development, reflecting a conservative decision-making orientation. Yet other studies demonstrate more positive effects. For instance, Hashai and Zahra [27] observed that international experience among founder teams initially enhanced firms’ technological expansion, though this effect diminished over time as reliance on existing knowledge created a competency trap. These findings reinforce the difficulty of isolating which kinds of professional experience advance or hinder technology development.

Beyond individual characteristics, broader institutional and organizational factors further complicate the picture. Yang et al. [17] showed that graduate education in China promoted technological development in high-tech firms but noted that this effect was stronger in private firms and in more developed regions. Barge-Gil et al. [18] highlighted that a high proportion of PhD-trained employees increased the likelihood of firms adopting upstream R&D strategies, yet this was neither a necessary nor sufficient condition for such a transition. Evidence from Solheim et al. [22] further suggests that contextual moderators, such as firm location, can determine whether accumulated worker experience leads to incremental technological development.

Some of this confusion may result from the fact that the data that previous studies have collected and analysed may be biased. First, the existing measures for technology development rely on indirect and subjective evaluation of technological input and output, such as R&D intensity and self-evaluation. Second, specific types of firms and industries have received considerable attention from scholars. Technology based firms, small and medium enterprises, and start-ups have been analyzed [12,28–30] since they are believed to be primary sources introducing technological innovation [31,32]. For the reason that high-tech industries put more emphasis on technologies and conduct more R&D activities [33], researchers chiefly build on new knowledge based on evidence from high-tech industries [7,34–36], thus neglecting other technological intensity levels (low, medium-low and medium-high).

In summary, current understanding of the role played by human capital (a key explanatory variable) on technology development (the phenomenon to be explained) is still limited by fragmented approaches, a dependence on indirect or subjective measures, and by the absence of any comprehensive analysis that controls for different industries, regions, types of firms, and types of human capital.

The objective of this study is to determine the extent to which human capital within a firm is associated with technology development. The research questions of this study are: (1) Do general and specific human capital affect technology development? and (2) How do general and specific human capital affect technology development jointly? For answering these research questions, we first create and employ an original and more objective measure of technological development based on companies’ transition from their current economic activity into a new one involving one with a higher or lower level of technological intensity. Second, we adopt a conceptual framework that directly links human capital with technological development and devote attention to the combined impact of different types of human capital. Third, to test our research hypotheses empirically, we use an extremely rich and unique longitudinal matched employer-employee microdata set – *Quadros de Pessoal* – that covers nearly all Portuguese private companies and their employees over a span of approximately 30 years. These provide a more precise and original approach that is more responsive to the whole gamut of forms of human capital and its impact on firms’ technology activities.

Portugal has strengthened its technological capabilities through integration within the European Union, where access to larger markets, collaborative research, and EU funding have supported opportunities for upgrading [37–39]. Human capital is central to this progress, supported by sustained investments in education and skills. Literacy and school participation are near universal, and educational attainment has steadily improved across generations. Increasing engagement in vocational and higher education, along with rising female labor force participation, further highlights Portugal’s growing capacity to build a skilled workforce for technology development [40]. Thus, Portugal provides a compelling context in which to study the relationship between human capital and technology development.

The results of our empirical analysis show that: 1) Education is an important form of general human capital which associates positively and significantly to technology development; 2) Industry experience is an important form of specific human capital which associates positively and significantly to technology development; and, 3) STEM education is a type of specific human capital that, by itself, does not suffice to explain technology development, but when combined with industry experience becomes important and associates positively and significantly with technology development.

In the following section, we review the literature regarding human capital and technological development and introduce five hypotheses. Section 3 explains the dataset used and the methodology. Section 4 presents the findings resulting from the analysis. Finally, based on the findings, Section 5 discusses policy implications and recommendations for firms and governments.

## Conceptual background and research hypotheses

### A new measurement of technology development

There is no universally agreed definition of technology development, and the literature reflects its multidimensional nature. Some scholars describe it as an integrated process of organizational and technological change, in which both organization and technology evolve together, guided by the practical requirements and needs identified by those directly engaged [41]. Others emphasize the importance of how firms manage and use resources in ways that support organizational expansion and profitability [42,43]. More recently, attention has been given to the equitable sharing of costs, benefits, and responsibilities among various stakeholders, including developers, promoters, government, industry, and users [44]. These perspectives reflect and emphasize that technology development can be understood in multiple ways, encompassing organizational integration, efficient resource use, and fairness in the distribution of outcomes across stakeholders.

This study develops and applies an original technology development measurement by tracking companies’ change of activity and technological development over time through the *Nomenclature of Economic Activities* (NACE). We apply an OECD’s typology which draws on the NACE, to classify firms into Low, Medium-low, Medium-high and High-tech levels [45,46] and assess whether a firm’s new activity has a higher (or lower) technological intensity.

The existing literature has measured the technological achievements of firms by using: firm survival [28,47]; number of employees [48]; firm sales [49]; size of funding [50,51]; number of patents [52], R&D intensity [52–55]; entrepreneurs’ self-evaluation [56–59]; or technical performance improvement [60]. These measures evaluate technological inputs and outputs indirectly. Thus, they have difficulty in clarifying whether the characteristics and efforts made by workers and managers’ have actually been associated with firms’ technological advancement. There have also been limitations in the measures used to assess the overall technological level of firms. Finally, some previous findings may be biased by the fact that they are based on responses to surveys in which subjective judgment may have played a major role [61] and may fail to cover important types of innovative activities [62,63].

By tracking changes in firms’ NACE codes, this study examines how human capital and other technological inputs are associated with transitions to higher technological intensity levels, thereby indicating meaningful technological progress. During the study period, Portuguese firms were required to register under a single primary NACE code defining their main business activity, and any modification to this classification signified a major structural transformation rather than an administrative update. Such reclassifications, formally validated by national authorities including the National Statistics Office (INE), the Tax Authority (AT), and the Institute of Registries and Notaries (RN), confirm that the firm has reoriented its core operations, technologies, or markets.

This makes it possible to observe real, market-acknowledged shifts in technological capabilities. Unlike traditional indicators that isolate specific aspects of innovation, the NACE-based approach captures firms’ ability to commercialize innovations and sustain new production systems over time. A transition to a new industry classification typically reflects workforce-driven learning, technological investment, and organizational adaptation, where employees acquire new competencies and integrate advanced processes to support higher-technology production [64–66]. Historical examples such as Honda’s evolution from motorcycle to automobile manufacturing, and Nintendo’s transition from playing cards to video games demonstrate how firms’ technological development manifests through such transformations. Accordingly, this measure provides a structural, dynamic, and organizationally grounded perspective on technology development capturing how human capital, learning, and innovation interact to produce long-term, commercially validated technological advancement.

A firm’s shift in industry classification reflects the culmination of internal technological advancement, encompassing the introduction and commercialization of products with greater technological complexity, resource intensity, and market sophistication. Commercialization plays a central role in this process, serving as the stage where technological potential is converted into economic value and where market feedback reinforces a firm’s technological capabilities [67,68]. Its success depends on organizational learning, which allows firms to acquire, assimilate, and apply internal and external knowledge to transform inventions into viable products. Through learning, firms develop capabilities, strengthen absorptive capacity, and integrate knowledge across organizational functions, thereby institutionalizing continuous improvement and sustained capability upgrading [67,68].

Human capital underpins this transformation by providing the foundation for learning and capability development. Employees’ skills, expertise, and creativity determine how knowledge is absorbed and converted into technological and commercial outcomes [69–71]. Well-developed human capital supports talent management and intellectual capital formation, while collective learning and a supportive knowledge-sharing climate facilitate the diffusion of knowledge across teams, reinforcing innovation and adaptability [69,71].

Empirical research substantiates these links: higher education fosters national and firm-level shifts toward high-technology activities, enhances absorptive capacity, and promotes restructuring through advanced product development and internationalization [72–74]. Together, this evidence demonstrates that firms with stronger human capital and learning systems are more capable of technological upgrading, making industry reclassification a meaningful indicator of organizational learning and capability enhancement.

### Human capital and technology development

Human capital, including the set of skills, knowledge, capabilities, and other attributes embodied in individuals, is widely acknowledged as a crucial determinant of technology development [75,76]. Numerous studies demonstrate that human capital consistently exerts a positive and statistically significant influence on technology development [77,78]. Therefore, firms, industries and countries often require highly skilled human capital, such as tertiary education attainment, to fully realize their technological potential [79]. Investments in human capital, typically through education and training, enhance the accumulation of skills, competencies, and knowledge that drive technological advancement [80].

The mechanism through which human capital fosters technology development is rooted in its role in building technological capabilities within firms [76,81]. Employees who engage in continuous technological learning contribute directly to the accumulation of these capabilities, enabling firms to sustain economic growth through innovation and productivity gains [82]. Human capital not only supports the development of technological knowledge but also enhances firms’ ability to absorb and apply new technologies, thereby facilitating adaptation and creation of novel solutions [79,83–86]. Accordingly, strategies aimed at strengthening human capital—particularly through human resource management (HRM) practices—have been shown to improve firms’ technological performance by reinforcing both knowledge acquisition and its effective utilization [87–89].

One stream in the literature addresses the relationship between human capital and technological development focusing on the concept of innovation. The results of these studies on innovation present mixed evidence, due to the existence of contextual differences among countries [90–92] and, eventually, because of the lack of a comprehensive conceptual understanding of how different human capital related internal and external factors affect, directly or indirectly, on firms’ technological trajectories [57].

It is useful here to divide the concept of human capital into two major categories: general and human capital that is specific to technology development [93,94]. General human capital is knowledge and skills that are transferable across jobs and industries whereas specific human capital is those that are most applicable within the specific contexts. This classification creates a more comprehensive nexus between the level of education, working experience, type of education, and industry experience among the firms’ workforce.

### General human capital and technology development

General human capital can be operationalized through measures of the level of formal education as well as labor market experience among workforce [95–97]. Previous literature has stressed the role of the educational attainment of employers on knowledge creation [98–100].

Better educated individuals may have project management skills such as setting better goals, a greater ability to motivate co-workers, and better communication skills [101,102]. Educational attainments and their interaction with varying expertise of employees lead to more dynamic communication, proactive knowledge integration, original and valuable idea creation [103]. Education contributes to improving the productivity of firms, which allows firms to devote more resources to technology development [100,104]. For example, higher levels of education can result in a higher probability of adopting a new technology that enhances a firm’s productivity. This effect of education is found to remain for an extended period of time after graduation [105]. Firms with more educated workforce are more likely to be funded by investors, because this is seen as signalling that firms have more entrepreneurial and technological capabilities [50,51]. Based on the empirical examination with technology ventures, Marvel and Lumpkin [106] show that education is positively influential to the likelihood of firms to enhance their innovative activities. Thus, firms which hire a large share of well-educated workers are likely to accelerate technology development. This leads us to advance:

*Hypothesis 1a*: The educational level of a firm’s workforce is positively associated with the firm’s transition to a higher level of technological intensity.

Individuals and teams with work experiences are found to have a positive impact on the technological performance of firms [107–110]. Workers who perform various functions across the firm may develop expanded understanding of technologies and build a more balanced view that incorporates different elements relating to technology development [13,111]. Prior work experiences may give workers more opportunities to accumulate knowledge about effective approaches to technology development through learning based on their prior successful and failed trials [112].

Work experience enhances managerial capabilities that are required to lead a group of employees or co-work with other entities. It can also enhance entrepreneurial abilities to recognize commercial opportunity [113]. Although technological knowledge obtained through education and experience is highly critical for a firm’s technological efforts, this technological knowledge can be linked to the performance of firms only when appropriate managerial and entrepreneurial skills are supported [113,114]. Otherwise, firms may excessively focus on technical dimension and make myopic decisions without thorough and comprehensive consideration about all feasible alternatives [114]. With evidence from a comparative case study of two R&D projects conducted by a firm in the lighting industry, Ravasi and Turati [115] note that experienced workers who tend to operate under serious resource scarcity are typically under considerable and constant pressure to enhance decision-making skills about what actions are worth pursuing to maximize expected outcomes, coordinate and integrates heterogeneous knowledge, skills and know-how. Furthermore, these potential strengths of technological and managerial expertise embedded in experienced workers have positive influence on financial support from external investors which is essential to increase R&D intensity [28,48,50,116]. With this in mind, we advance the following hypothesis:

*Hypothesis 1b*: The work experience level of a firm’s workforce is positively associated with the firm’s transition to a higher technological intensity level.

### Specific human capital and technology development

Much literature has accepted the definition of specific human capital as an individual’s capabilities that are particularly relevant to the business or job concerned [13,116–118]. Within the context of a firm’s transition to a higher technological intensity level, and following Henning et al. [116] and Stucki [94] categorization, this paper conceptualizes specific human capital as the specific education and experience closely related to and demanded for technology development in firms.

Employees who have academic degrees related to engineering and science fields are likely to have a higher level of understanding of the technologies that firms are applying or attempting to develop. It is not surprising that the employees with such expertise help firms to create more technologically advanced products. The level of STEM education contributes to technological development through two mechanisms: increased ability to recognize and value external technologies; and, the facilitation of internal technology integration and generation [119]. These two mechanisms are aligned with the concept of absorptive capacity which is comprehensively conceptualized and elaborated by Cohen and Levinthal [120]. Jiménez-Barrionuevo et al. [121] define absorptive capacity as “the organization’s relative ability to develop a set of organizational routines and strategic processes through which it acquires, assimilates, transforms and exploits knowledge acquired from outside the organization in order to create value”. The empirical literature on technology based firms reports a positive impact of the technical and scientific education on technological performance of firms [11,48,52,113,122]. Therefore, we advance the following hypothesis:

*Hypothesis 2a*: The STEM education level of a firm’s workforce is positively associated with the firm’s transition to a higher technological intensity level.

It is likely that employees who have gained a significant stock of knowledge, skills and techniques in the same industry play an important role in a firm’s undertake technology development [11]. Training programs in the workplace enhance manufacturing workers, engineers and managers do specific jobs in a better way [123–125]. Individuals who have work experience in the same sector have a better understanding about industry-related technologies and are more likely to discover technological opportunities, search for required resources, and participate actively to set up and shape a firm’s knowledge strategy [126]. Moreover, empirical studies investigating international labor migration imply that workers who develop a stock of knowledge in the same industry are likely to be able to contribute to technology spillover between firms [101,127,128]. In line with these findings, we expect that firms having employees with more industry specific experience are likely to make more technological progress and move to technologically advanced industry. Hence, we put forward the following hypothesis:

*Hypothesis 2b*: Specific industry experience of a firm’s workforce is positively associated with the firm’s transition to a higher technological intensity level.

Individuals may obtain prerequisite technological knowledge and skills through a STEM curriculum that provides basic competency for conducting activities within a firm. Although the improvement in human capital through education and its importance in technology development is well-acknowledged, there are studies documenting that educational attainment is not directly linked to undertaking given tasks and taking lead roles in firms’ technology development [129–133]. According to Hayter and Parker [133], many fresh STEM degree holders have considerable difficulties in understanding how the real world works and applying their knowledge and skills in support of technological activities of firms. There is often a mismatch between skills that students learn in their scientific education and training, and those required to pursue job responsibilities [131,134,135]. In particular, when individuals with specialties outside the scope of the firms’ current technological outlook are hired, this mismatch is more common and apparent [136]. Thus, graduates often lack the required skills to transfer their scientific and engineering knowledge into engineering model and practices in the context of commercial opportunity [130,133]. Furthermore, much STEM education tends to underestimate the importance of social and communication skills such as presentation and interaction with diverse workers [131].

Experience in the same industry helps workers develop educated expertise on real-world projects [137]. The involvement in executing tasks to develop technologies in a firm offers practical knowledge and skills that educational institutions pay less attention to [132]. Furthermore, it provides the latest technologies in the industries to workers [138,139]. On the job training leads to impart firm-specific knowledge that can be applied in the specific environment of firms and increase the productivity of workers [101]. Findings in this literature suggest the following hypothesis:

*Hypothesis 3*: The existence of individuals endowed both with STEM education *and* specific industry experience within a firm’s workforce positively associates with the firm’s transition to a higher technological intensity level.

**Fig 1:**
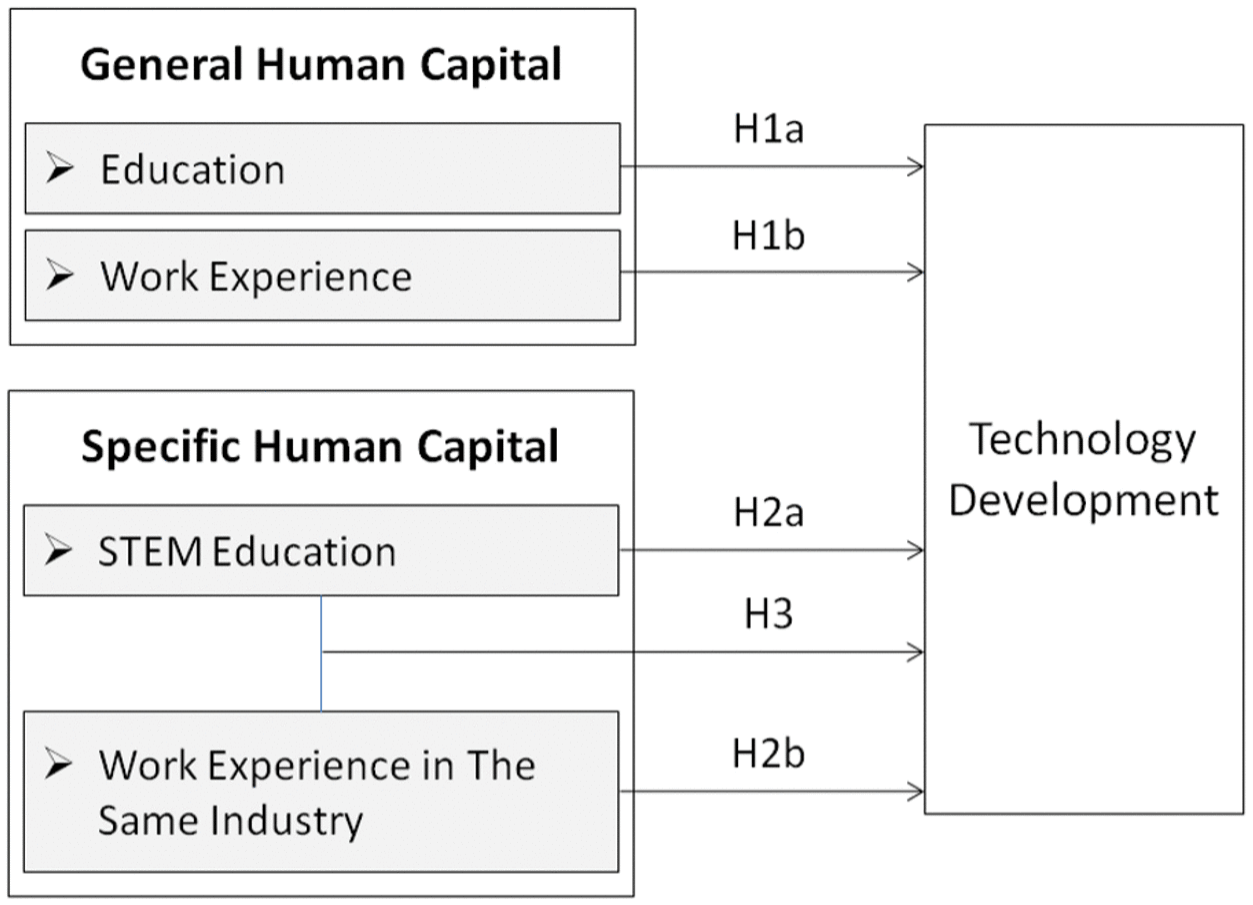
Research model.

In summary, Fig 1 shows the hypotheses tested in this study.

## Dataset and methods

### Dataset

Our empirical investigation draws on an extremely rich and unique matched employer-employee microdata set – *Quadros de Pessoal (QP)*. The QP, which is made accessible to authorized users under legal and regulatory protections, was accessed, collected, and analyzed throughout the entire research period, beginning on April 30, 2019, and extending to May 8, 2025. There is no collection, analysis, or linkage of individual-level private information, and the research does not involve any form of intervention or interaction with human subjects.

The QP covers a span of roughly 30 years (1985–2015). With the exception of public administration, nearly all public/private companies which hired at least one employee/wage-earner in either Continental Portugal or in Autonomous Regions (islands), together with their employees, are included in these data. The QP survey is conducted by the Portuguese Ministry of Social Security and Labour and participation is mandatory for all employer firms (reporting is not a legal obligation for sole traders). This guarantees coverage of all relevant incorporated businesses in our data.

The fact that *Quadros de Pessoal* allows for tracking nearly all firms, industries, intensity levels and firm’s workforce in the country over the course of a long period, makes it the ideal source of data for investigating the relationship between human capital and technological development of firms. There are around 200,000 firms and over 2.5-million workers in each annual survey. The longitudinal matched employer-employee data include extensive information on the mobility of firms, paid employees and business owners. Individuals and firms are fully linked and can be tracked over time using a unique identification number.

The data used in this research consist solely of firm-level aggregated information. The dataset includes aggregated information on organizational characteristics such as demographics, industry classification, financial indicators, ownership structure, performance metrics, and geographic location. It also includes aggregated summaries of employee characteristics at the establishment level, including average demographic attributes, distributions of occupational roles, compensation metrics, working hours, and indicators of human capital. The dataset contains no personal identifiers, nor does it include any variables that could be used to re-identify individual employees either directly or indirectly.

The first and second version of CAE/NACE codes which were used during 1985 ~ 2008 are not detailed and are substantially different from subsequent versions. This hampers the harmonization of all the information over the time covered by the dataset. Therefore, we filtered out previous years and focused on data from 2009 to 2015 (CAE Rev.3).

The present analysis focuses on the concept of technology intensity, which connects with firms operating exclusively in manufacturing industries. Accordingly, this empirical research considers firms with their first 2-digits CAE/NACE codes starting from 10 (food and beverages manufacturing) to 33 (Others manufacturing). The number of firms in manufacturing industries accounts for, approximately, 10% of all the observations in 2009–2015. This proportion is aligned with the report from Instituto Nacional de Estatística (the National Institution of Statistics) of Portugal and the rest of the data mainly consists of the service providers of various sectors including commerce (23%), construction (19%), accommodation and restaurant (10%), transportation (5%), and others (26%) [140]. Excluding firms due to missing data, we ended up with a dataset of 80,995 observations.

### Variables

#### Dependent variable.

The technology development of firms is measured via the change of the reported technology intensity level of each firm. Portuguese firms which respond to the survey mark their CAE code depending on the specificity of their main economic activity. Economic activity covers a range of resource input, production process, and product output within the firm. We classified the CAE codes into four technology intensity levels following the definition by the OECD [45] and Eurostat [46]; low-tech, medium low-tech, medium high-tech and high-tech industries. For example, the pharmaceutical industry is classified as a high-tech industry whereas rubber and plastic manufacturing is in medium low-tech industry. By tracking firms’ transitions between CAE codes according to the four technology intensity levels, the technology development of firms can be identified, evaluated and tracked. The dependent variable is a dummy variable which takes the value ‘1’ if a firm moves to an industrial activity with higher technology intensity level. A transition into a higher technological level is path dependent and it is usually the lagged outcome of an assortment of organizational inputs, resources, strategies and behaviours available/established in previous years [141]. Therefore, we investigate the effect that a set of independent variables at a given year *t* produces on the firm technology development at year t + 2. A two-year time lag model is suitable to the phenomenon under analysis and widely accepted in the literature [142,143].

#### Independent variables.

The first group of variables of interest is used to investigate the role of firms’ general human capital and consists of education level and work experience. The overall education of employees is measured in terms of eight levels based on the number of years of schooling regulated by the Ministry of Education in Portugal [144,145]: (1) Primary education (6 years of education) or less; (2) Lower secondary education (3 years); (3) Upper secondary education (3 years in academic or practical courses); (4) Post-secondary education (professional training mainly required for nurses and elementary school teachers); (5) Bachelor’s degree; (6) Post-graduate degree (specialized education mainly for bachelor’s degree holders); (7) Master’s degree; and (8) Ph.D. Work experience is measured by the number of years worked. For the analysis, we use the firm-level averages of these two variables, workforce education and work experience.

The second group refers to the specific human capital in firms. This group includes STEM education and work experience in the same industry. STEM education is measured by a ratio of workers having STEM related educational background [146–148]. Work experience in the same industry is included as the average number of years that workers experienced in the same industry (where he/she works presently) before being enrolled in their current firm [19,149]. To investigate the synergetic gains between these two specific human capital variables, an interaction variable combining STEM education and industry work experience was developed. The combination variable of STEM education and work experience in the same industry is a ratio of workers with STEM education and work experience in the same industry. This variable is set equal to 1 if a worker has both a STEM degree and same industry work experience, otherwise it is set equal to 0.

#### Control variables.

Drawing on prior studies, we employ several control variables in our empirical analyses. First, we include foreign ownership, as it may impact a firm’s preference for human capital [150,151]. This variable is measured as the percentage of the foreign owned capital in the firm. Second, we control for initial capital (in euros) as, for example, according to Honjo [30], laborers with technological capabilities are closely related to the size of initial capital and initial financing size has an impact on firm performance [152,153]. Third, the type of ownership, such as private and public ownership, may affect technology development activities [55,154]. Hence, each firms’ ownership type is included as a control and measured as the ratio of private capital to total assets in a firm. Fourth, we include information on firm sales as this may influence the amount and span of a firm’s resources that are available to be invested for technology development [155]. Fifth, we control for firm size because the number of workers can be closely linked with the firms’ ability to search, absorb and employ external technologies that are critical for new product development [156]. Firm size can also affect a firms’ productivity [157]. Measure of firm sales and size are operationalized using ten quantiles to address the skewed nature of the distribution. Finally, this study introduces region, municipality, firm size and firm age dummies for the additional analysis. The region dummies, which consist of Norte, Lisbon Metropolitan Area, and Algarve regions of Portugal, are equal to 1 if a firm is located in the corresponding region. The municipality dummies take the value of 1 if a firm is located in the biggest city of each region that is Porto, Lisbon, and Faro respectively. The firm size dummies are variables categorizing the firm size following the EU’s classification of firms [157,158]. They include micro (less than 10 employees), small (less than 50 employees), medium (less than 250 employees) and large sizes. The firm age dummy is equal to 1 if a firm is older than 6 years [159,160].

### Estimation method

A logistic regression model was estimated to test our hypotheses. The logistic regression model is appropriate to the purpose of this study to understand the relative likelihood of a firm ascending the ladder of technology intensity levels. This statistical technique is appropriate to the data under study since the dependent variable has binary responses and the independent variables are continuous. We report the coefficients indicating the probability of the firm climbing the ladder of technology development among ‘low-tech’, ‘medium low-tech’, ‘medium high-tech’ and ‘high-tech’ industries. To make it easier to interpret the outcomes of the logistic regression we also report marginal effects, which give us a measure of the effect that each independent variable has on the probability. The marginal effect is the probability that the respective category of the dependent variable changes when the independent variable increases by 1 unit, fixing all the other variables at the mean values [161].

The descriptive statistics are presented in Table 1. Among all 80,995 firm-year observations, 60.49 percent belong to low technology industries, 30.98 percent to medium low technology industries, 7.82 percent to medium high technology industries, and only 0.73 percent to high technology industries. This distribution shows that most Portuguese firms operate in low or medium low technology sectors and that advancing to higher technology levels is progressively more difficult. During the period 2009–2015, only 0.25 percent of Portuguese firms (i.e., 202 firms) transitioned to industries with a higher technological intensity level, with this proportion fluctuating between 0.15 percent and 0.51 percent across years. This variation suggests that Portuguese firms were gradually recovering from the economic recession, progressively regaining their capacity to invest in technological upgrading and structural transformation. This finding confirms that structural technological upgrading is a complex, demanding, and relatively rare process. The distribution of firm sales and employment is highly skewed to the right, indicating that the majority of Portuguese firms remain relatively small in size. When compared with firms that did not experience a change in technological intensity, the 202 transitioning firms employ slightly fewer workers but achieve similar levels of annual sales, suggesting higher labor productivity and a greater focus on value added activities. In the tenth quantile distribution, transitioning firms record an average workforce score of 5.871 compared with 6.183 for non-transitioning firms, while their sales scores are nearly identical at 6.149 and 6.205, respectively. These characteristics support the interpretation that industry transition reflects a structural, knowledge driven transformation grounded in capability enhancement rather than a mere increase in size or output. Among the transitioning firms, approximately half (119 firms) moved from medium-low-technology to medium-high-technology industries, while only a few, specifically seven firms, advanced from medium-high-technology to high-technology industries. This pattern implies that firms with an established technological base, such as those operating in medium low technology industries, are particularly well positioned to progress toward higher technological levels because they already possess the necessary production capabilities, accumulated knowledge, and absorptive capacity to support further upgrading. Conversely, firms in low technology industries, despite representing the majority of the overall population, account for a smaller proportion of successful transitions. This suggests that the absence of a technological foundation and limited capability accumulation severely constrain their ability to undertake complex technological development. Furthermore, this distribution reflects the structural reality that developing and commercializing high-technology products and successfully entering high-technology sectors remains exceptionally challenging. In Table 2, we provide the correlation matrix of the variables. Most correlation values among the variables are relatively small, thereby suggesting that the risk of multicollinearity is low. The Variance Inflation Factor (VIF) test was also run and shows the absence of collinearity among the variables.

**Table 1:** Variables and descriptive statistics.

Variables	All firms (N = 80,995)				Transitioned firms (N = 202)				Non-transitioned firms (N = 80,793)			
	Mean	S.D.	Min	Max	Mean	S.D.	Min	Max	Mean	S.D.	Min	Max
*Independent Variables*												
General Human Capital												
Education level	2.473	0.826	1	8	2.735	1.077	2	7	2.473	0.825	1	8
Work experience (years)	2.251	0.959	1	12	2.221	1.024	1	9	2.252	0.959	1	12
Specific Human Capital												
STEM education (%)	0.024	0.100	0	1	0.033	0.104	0	0.833	0.024	0.100	0	1
Work experience in the same industry (years)	3.158	2.114	1	15	3.738	2.480	1	10	3.157	2.112	1	15
STEM education and Work experience in the same industry	0.131	0.337	0	1	0.173	0.379	0	1	0.131	0.337	0	1
*Control Variables*												
Foreign capital (%)	2.025	13.666	0	100	0.495	7.036	0	100	2.029	13.679	0	100
Initial capital (Millions of Euros)	0.482	14.711	0	2,000	0.210	0.987	0	11.1	0.482	14.711	0	2,000
Private capital (%)	80.529	39.436	0	100	85.644	35.152	0	100	80.516	39.445	0	100
Firm sales (Millions of Euros)	6.205	2.845	1	10	6.149	3.048	1	10	6.205	2.844	1	10
Number of workers	6.182	3.109	1	10	5.871	3.091	1	10	6.183	3.109	1	10

**Table 2:** Correlation matrix of the independents and control variables.

	Variables	1	2	3	4	5	6	7	8	9	10
1	Education level	1.00									
2	Work experience	−0.03^***^	1.00								
3	STEM education	0.48^***^	0.00	1.00							
4	Work experience in the same industry	0.11^***^	−0.08^***^	0.02^***^	1.00						
5	STEM education and Work experience in the same industry	0.33^***^	0.02^***^	0.61^***^	0.00	1.00					
6	Foreign capital	0.12^***^	0.03^***^	0.11^***^	−0.00	0.23^***^	1.00				
7	Initial capital	0.04^***^	−0.00	0.02^***^	0.00	0.06^***^	0.05^***^	1.00			
8	Private capital	0.06^***^	0.09^***^	0.03^***^	0.03^***^	0.06^***^	−0.28^***^	−0.01^*^	1.00		
9	Firm sales	0.08^***^	0.07^***^	0.09^***^	−0.02^***^	0.35^***^	0.15^***^	0.04^***^	0.30^***^	1.00	
10	Number of workers	−0.01	0.11^***^	0.05^***^	−0.10^***^	0.34^***^	0.13^***^	0.04^***^	0.31^***^	0.74^***^	1.00

*** p < 0.001, ** p < 0.01, * p < 0.05.

## Results

### Estimation results

Our five hypotheses were tested using logistic regression models. The results are presented in Table 3. Model 1 with four independent variables served as the baseline and Model 2 added the interaction term between STEM education and work experience in the same industry. All the models controlled for firms’ characteristics, such as firm size and capital structure, and included the constant term. The constant term is not reported in the table of results.

**Table 3:** Logistic regression analysis predicting firms’ technology development (N = 80,995 engaged in manufacturing).

Variables	Model 1		Model 2	
	Coef.	*Std.Err.*	Coef.	*Std.Err.*
*Independent Variables*				
* *General Human Capital				
* *Education level	0.258^***^	0.071	0.251^***^	0.071
* *Work experience	−0.002	0.075	0.000	0.075
* *Specific Human Capital				
* *STEM education	−0.226	0.587	−1.231	0.817
* *Work experience in the same industry	0.093^**^	0.030	0.092^**^	0.030
* *STEM education and Work experience in the same industry			0.600^*^	0.276
*Control Variables*				
* *Foreign capital	−0.014	0.011	−0.015	0.011
* *Initial capital	0.000	0.000	0.000	0.000
* *Private capital	0.003	0.002	0.003	0.002
* *Firm sales	0.019	0.038	0.009	0.038
* *Number of workers	−0.035	0.035	−0.051	0.036
*Model summary*				
* *Pseudo R^2^	0.012		0.014	

*** p < 0.001, ** p < 0.01, * p < 0.05.

The first set of the hypotheses (*Hypothesis 1a* and *1b*) state that general human capital of workers in a firm increases the possibility of technology development, stressing the importance of education level and work experience. Our results show higher possibility of technology development when firms possess more educated workers on average (*b* = 0.258, *p* < 0.001), thereby supporting *Hypothesis 1a*. The marginal effect analysis indicates that a one standard deviation increases in the average level of education (0.826) results in a 0.054 percentage point (pp) increase in the likelihood of firms moving up in the ladder of the technology intensities. Although this increase may seem very small, it is substantial in light of the fairly low rate at which Portuguese firms move to a higher technology intensity level (0.25%). This finding aligns with the literature highlighting that workers with more education may have better technological and managerial capabilities to engage in more effective and efficient technology-related activities, such as product development, problem solving, communication and opportunity detection within the firm.

The coefficient between general work experience and technology development is not statistically significant; therefore, we do not confirm *Hypothesis 1b* that the work experience level of a firm’s workforce alone is positively associated with the firm’s technological development.

With regard of specific human capital, the analysis does not support *Hypothesis 2a*, which posit that STEM education leads to greater likelihood of technology development for firms. The results show that, by itself, a workforce endowed with STEM education is not significant in stimulating a firms’ technology development.

However, *Hypothesis 2b*, which expected a positive impact of work experience in the same industry on technology development, is confirmed. The coefficient of industry experience is positive and significant as proxied by number of studies (*b* = 0.093, *p* < 0.01). A one standard deviation increases in the average years of work experience of workers the same industry (2.114) is associated with a 0.049 pp increase in the probability of technology upgrade. This magnitude is approximately 20% of the predicted possibility of technology development.

We previously discussed the conceptual and empirical importance of combining STEM education with industry experience on technology development. According to *Hypothesis 3*, the existence of individuals endowed both with STEM education *and* specific industry experience within a firm’s workforce positively associates with the firm’s technological development. The coefficient of the variable interacting STEM education and Industry experience in Model 2 is positive and significant (*b* = 0.600, *p* < 0.05). Consequently, Hypothesis 3 is supported, underlining the importance of this mediating effect on firms’ propensity to move to the higher technology intensity level. Holding other variables at their mean, a 1% increase in the percentage of workers with STEM education *and* industry experience leads to a 0.0015 pp increase of the likelihood of technology development. In other words, the probability to move up to a technologically more intensive industry category increases by 0.05 pp given a one standard deviation increase in the rate of workers who have STEM education *and* the prior work experience in the same industry occurs. This change is nearly 22% of the average probability of a firm climbing up in the technology intensity ladder.

**Fig 2:**
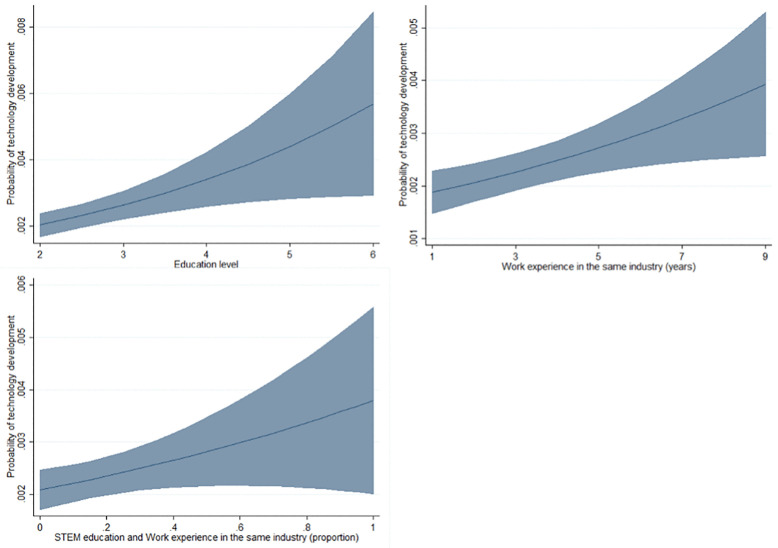
Predictive margins of general and specific human capital on technology development.

Fig 2 maps the predictive margins of education, industry experience and the combination of STEM education and industry experience on technology development based on the regression coefficient from the analysis. The graphs clearly show that all three variables are statistically significant and positive across the distribution of each variable as predicted in Table 3.

In summary, the following main outcomes can be emphasized: 1) Education is an important form of general human capital which associates positively and significantly to technology development; 2) Industry experience is an important form of specific human capital which associates positively and significantly to technology development; and, 3) STEM education is a type of specific human capital that, by itself, is not suffice to explain technology development but when combined with industry experience becomes important and associates positively and significantly to technology development.

The R-squared values of the models are small. First, this indicates that there could be a number of relevant factors that were not observed or considered, or there could be considerable heterogeneity in the firms in the data set. Scholars suggest that a low R-squared value in some fields like social science is inevitable and common since there are inherently unexplainable dimensions in human behaviour [162,163]. Indeed, it is not surprising that the explained variance remains modest, since technological development is likely influenced by a wide range of direct and indirect variables. In the context of this study, possible candidates include individual-level characteristics such as self-efficacy and autonomy; firm-level attributes such as gender diversity, top management team diversity, R&D efforts, IT adoption, internationalization strategies, foreign trade orientation, and the age structure of the workforce; and socio-economic factors such as income levels, demographic composition, and infrastructure. Additional influences may also arise from institutional and policy environments, including regulatory frameworks, government incentives, and intellectual property protections, as well as from cultural dimensions such as national attitudes toward risk-taking and innovation, and industry-level dynamics such as competitive intensity and knowledge spillovers. Second, the rare occurrences of the transition among different technology intensities (0.25%) could bias the estimation and possibly lower the R-squared value of our models [164]. Furthermore, this study analyses the whole population covering most of firms and employees in Portugal. Thus, our R-squared values are not estimates from samples that are likely to be biased by many external factors, but the R-squared value of the population, which is meaningful to understand the influence of human capital on technology development.

### Additional analysis

As additional analysis, we tested whether the differences among the human capital factors that may influence technology development are related to municipality and regional engagement. This study assessed the regional activities of Norte, Lisbon Metropolitan Area, and Algarve regions among all the eight regions of Portugal categorized by the Nomenclature of Territorial Units for Statistics 2 (NUTS2) of European Statistical Office (Eurostat). Five other regions served as reference category. Norte and Lisbon Metropolitan Area regions have been the most industrialized regions in the history of Portugal whereas the Algarve region has gained more recent attention from investors based on its digital industry [165,166]. These three regions hold 68.2% of the total number of firms and 73% of employees in Portugal in 2019 [167–169]. Porto, Lisbon and Faro are the largest cities of these regions functioning as the centers of the regional economies.

We included the municipality dummies in Model 3 and 4 and the regional dummies in Model 5 and 6. Table 4 presents the estimation results of those models. The results reconfirm the positive relationship of education, industry experience and the combination of those two human capitals on technology development. The differences in the marginal effects between the models that include municipality dummies, and the original models, are trivial. However, the marginal effects for the statistically significant variables become weaker by 3%, 22%, and 7% respectively when we add the region dummies. In addition, we find differences in the possibility of technology development among the regions. Firms in the Norte region show a considerably lower likelihood of moving to higher technology intensity levels (approximately 67% lower than a reference category). One potential reason for observing fewer transitions of firms in the Norte region may be that this region is the home of traditional industries such as the textile, apparel, footwear, food and furniture industries. The industrial clusters of the fashion industry contribute 11% of the gross value added (GVA) created in Norte region [165] whereas the proportion of the GVA from technology-based products is less than 1% that is substantially lower than the average of Portugal [166].

**Table 4:** Logistic regression results with region and municipality dummy variables.

Variables	Model 3		Model 4		Model 5		Model 6	
	Coef.	*Std.Err.*	Coef.	*Std.Err.*	Coef.	*Std.Err.*	Coef.	*Std.Err.*
*Independent Variables*								
*** ***General Human Capital								
*** ***Education level	0.263^***^	0.071	0.255^***^	0.072	0.248^***^	0.072	0.241^***^	0.072
*** ***Work experience	−0.004	0.075	−0.002	0.075	−0.010	0.075	−0.009	0.075
*** ***Specific Human Capital								
*** ***STEM education	−0.228	0.588	−1.232	0.817	−0.198	0.592	−1.130	0.817
*** ***Work experience in the same industry	0.090^**^	0.030	0.089^**^	0.030	0.071^*^	0.030	0.071^*^	0.030
*** ***STEM education and Work experience in the same industry			0.598^*^	0.276			0.553^*^	0.276
*Control Variables*								
*** ***Foreign capital	−0.014	0.011	−0.015	0.011	−0.014	0.011	−0.015	0.011
*** ***Initial capital	−0.000	0.000	0.000	0.000	−0.000	0.000	−0.000	0.000
*** ***Private capital	0.003	0.002	0.003	0.002	0.003	0.002	0.003	0.002
*** ***Firm sales	0.019	0.038	0.009	0.038	0.008	0.038	−0.001	0.038
*** ***Number of workers	−0.036	0.035	−0.052	0.036	−0.025	0.035	−0.040	0.036
*** ***Municipality – Baseline ‘Others’								
*** ***Porto	−0.567	1.005	−0.568	1.005				
*** ***Lisbon	−0.373	0.715	−0.368	0.715				
*** ***Faro	–		–					
*** ***Region – Baseline ‘Others’								
*** ***Norte					−0.682^**^	0.242	−0.662^**^	0.242
*** ***Greater Lisbon					−0.234	0.304	−0.226	0.304
*** ***Algarve					–		–	
Pseudo R^2^	0.013		0.014		0.015		0.017	
Observations	80,866		80,866		80,021		80,021	

*** p < 0.001, ** p < 0.01, * p < 0.05.

To examine whether the impact of human capital was associated with the different size groups of firms, we used firm size dummies consisting of micro (less than 10 employees), small (less than 50 employees), medium (less than 250 employees) and large sizes. Results of Model 7 and 8 reported in Table 5 are stable and similar to the previous analysis with the approximately 3% increased marginal effects. In addition, the results on large sized firms are positive and significant. It indicates that large firms are more likely to develop technologies and expand their business to technologically more intensified one. The results demonstrate that technology development relies on different resources and organizational processes, which might be limited and affected by firm size. This is in line with the recent studies which found that larger firms are better positioned to access resources, overcome managerial constraints, efficiently conduct technological activities and succeed in technology development [170–172].

**Table 5:** Logistic regression results with firm size and firm age dummy variables.

Variables	Model 7		Model 8		Model 9		Model 10	
	Coef.	*Std.Err.*	Coef.	*Std.Err.*	Coef.	*Std.Err.*	Coef.	*Std.Err.*
*Independent Variables*								
*** ***General Human Capital								
*** ***Education level	0.267^***^	0.071	0.261^***^	0.071	0.261^***^	0.071	0.253^***^	0.071
*** ***Work experience	−0.006	0.075	−0.004	0.075	0.006	0.075	0.007	0.075
*** ***Specific Human Capital								
*** ***STEM education	−0.236	0.594	−1.166	0.845	−0.219	0.588	−1.220	0.817
*** ***Work experience in the same industry	0.096^**^	0.030	0.095^**^	0.030	0.088^**^	0.030	0.088^**^	0.030
*** ***STEM education and Work experience in the same industry			0.557	0.300			0.596^*^	0.276
*Control Variables*								
*** ***Foreign capital	−0.017	0.011	−0.018	0.011	−0.014	0.011	−0.015	0.011
*** ***Initial capital	−0.000	0.000	−0.000	0.000	−0.000	0.000	−0.000	0.000
*** ***Private capital	0.003	0.002	0.003	0.002	0.003	0.002	0.003	0.002
*** ***Firm sales	−0.004	0.032	−0.013	0.033	0.015	0.038	0.005	0.039
*** ***Firm size – Baseline ‘Micro’								
*** ***Small	−0.062	0.204	−0.153	0.212				
*** ***Medium	−0.128	0.400	−0.398	0.431				
*** ***Large	1.791^**^	0.616	1.450^*^	0.645				
*** ***Firm age – Baseline ‘Young’								
*** ***Mature					0.169	0.260	0.158	0.260
Pseudo R^2^	0.014		0.016		0.012		0.014	
Observations	80,995		80,995		80,995		80,995	

*** p < 0.001, ** p < 0.01, * p < 0.05.

Firm age may affect the probability of technological growth of firms. Many scholars have focused on the relationship between firm age and technological output [160,173–175]. To explore whether our results are linked to the effect of firm age, the original estimation was repeated with the firm age dummies. The results shown in Model 9 and 10 of Table 5 are similar to previous ones, although the marginal effect of the industry experience becomes smaller by approximately 4%. There is no evidence of a positive or negative association between firm age groups and technology development. Mature firms, which are older than 6 years, show positive coefficients, however, they are not statistically significant. This suggests that technological advancement including R&D output and commercialization is arduous regardless of firm age. Moreover, compared to the previous analysis of firm size, our results imply that firm-level resources including R&D capabilities and managerial skills that are critical in technology development are more likely to be influenced by firm size rather than firm age.

### Robustness check

We complemented the analysis with additional tests to evaluate the robustness of the results. Our data show that the transition of firms within the different levels of technology intensity, which is the dependent variable of this study, is a relatively rare event. According to King and Zeng [164], logistic regression with rare events data can be biased towards the majority cases and underestimate the probability of rare events. To consider the concerns about potential biases caused by the rareness of these events, we adopted the Rare Events Logistic Regression and Firth Logistic Regression. The Rare Events Logistic Regression applies a bias corrected estimator and hence generates a lower mean square error for coefficient and possibilities [164] whereas the Firth Logistic Regression uses a penalized maximum likelihood estimation method to minimize bias known as “rare event bias” or “small sample bias” [176–178]. The consistent results represented in Table 6 support the robustness of our models. In addition, the Chi-squared values and the penalized log likelihood of the Firth Logistic Regression show that the model change is statistically significant and the model fit is improved by adding the independent variable. To check the robustness of our findings in the additional analysis, we ran the same regression with the sliced data following the criterion mentioned in the previous section. The results obtained stay generally consistent with those displayed and discussed herein.

**Table 6:** Robustness check with rare events logistic regression models (‘relogit’ and ‘firthlogit’ in STATA).

Variables	relogit				firthlogit			
	Model 11		Model 12		Model 13		Model 14	
	Coef.	*Std.Err.*	Coef.	*Std.Err.*	Coef.	*Std.Err.*	Coef.	*Std.Err.*
*Independent Variables*								
* *General Human Capital								
* *Education level	0.262^***^	0.072	0.256^***^	0.073	0.262^***^	0.070	0.254^***^	0.071
* *Work experience	0.003	0.080	0.005	0.080	0.002	0.074	0.004	0.074
* *Specific Human Capital								
* *STEM education	−0.149	0.474	−1.052	0.662	−0.154	0.571	−1.033	0.781
* *Work experience in the same industry	0.093^**^	0.031	0.093^**^	0.031	0.093^**^	0.030	0.092^**^	0.030
* *STEM education and Work experience in the same industry			0.562^*^	0.262			0.541^*^	0.269
*Control Variables*								
* *Foreign capital	−0.010	0.012	−0.011	0.012	−0.010	0.009	−0.012	0.009
* *Initial capital	0.000	0.000	0.000	0.000	0.000^*^	0.000	0.000^*^	0.000
* *Private capital	0.003	0.002	0.003	0.002	0.003	0.002	0.0028	0.002
* *Firm sales	0.017	0.041	0.008	0.041	0.017	0.038	0.008	0.038
* *Number of workers	−0.038	0.035	−0.052	0.037	−0.037	0.035	−0.052	0.036
Degrees of freedom	9		10		9		10	
Penalized log likelihood					−1345.00		−1341.80	
χ^2^					45.05^***^		48.20^***^	
Observations	80,995		80,995		80,995		80,995	

*** p < 0.001, ** p < 0.01, * p < 0.05.

## Discussion and conclusions

Prior research about human capital and technology development assessed technology development of firms through potentially biased measures, originating from subjective self-reported surveys, or indirect evaluation, such as R&D intensity. For example, the usefulness of R&D intensity, one of the most widely used indicators to explain technological capability, is of limited value since it is not possible to reflect non-R&D factors that are a critical element to enhance technological capabilities [179]. This study proposes an original measure that can more adequately capture the overall (static) technological level of firms at a given moment in time and firms’ (dynamic) structural change based on a transition from their current business into a new one that involves a higher technological intensity level.

Our results advance the knowledge about human capital by reinforcing the finding that the educational level of a firm’s workforce is positively associated with the firm’s transition to a higher level of technological intensity (Hypothesis 1a). Prior studies indicate that educational attainments and their interaction with varying expertise among employees facilitate dynamic communication, proactive knowledge integration, and the creation of original and valuable ideas [180].Education also improves firm productivity, enabling companies to devote more resources to technology development [86,181]. This highlights the broader role of education in strengthening absorptive capacity and technology adoption, and it underscores the value of investing in a more educated workforce to accelerate technological upgrading and enhance competitiveness in technology-intensive industries.

In the realm of specific human capital, our results show that prior same-industry work experience has a positive and significant effect on technology development (Hypothesis 2b). This finding complements earlier research showing that individuals with industry-specific experience have a more sophisticated understanding of relevant technologies, are better positioned to discover technological opportunities, and actively contribute to shaping a firm’s knowledge strategy [182]. Beyond confirming that human capital is multidimensional, the result suggests that workers with same-industry experience can provide tacit knowledge and routines critical for upgrading technologies. For firms, this points to the strategic importance of recruiting or retaining employees with deep industry backgrounds to enhance their capacity for technology development.

Interestingly, our findings indicate that STEM education alone is not significantly associated with technological development but becomes positively linked when complemented by prior same-industry experience (Hypothesis 3). This resonates with research showing that many fresh STEM graduates encounter difficulties in applying their knowledge to real-world technological activities, often due to mismatches between academic training and workplace demands [183–185]. The problem is especially evident when graduates’ specialties diverge from a firm’s technological scope [186]. As a result, STEM graduates may lack the practical ability to translate scientific and engineering knowledge into commercially viable practices [187,188]. Industry-specific experience mitigates this gap by providing hands-on expertise through real-world projects [189], task-based learning within firms [190], and exposure to the latest industry technologies [191,192].

These findings highlight the complementarity between formal education and experiential learning, and they imply that stronger pathways for integrating STEM graduates into firms are essential for maximizing the impact of STEM education on technological development. Many firms, particularly in the knowledge-intensive industries (services), attempt to hire STEM workers including Ph.D. students who have just received their degree. Our results indicate that STEM educated employees are not always advantageous to firms that engage in technology development. Given the findings that the benefit of STEM education can arise from same-industry work experience, it is imperative that firms pay attention to how they build the composition of their workforce with employees who received degrees in STEM related majors *and* have work experience in the same industry as well. Otherwise, firms may have to invest in internal programs that complement and balance these workers’ specific education with specific practical technological and operational skills.

Moreover, the empirical results imply that firms need to elevate the overall education level of employees. Well-educated workers may shape a more supportive work environment for technology development by engaging in effective communication and leadership. Firm managers should be cautious about entertaining equilibrium among these three major indicators of human capital – general education level, STEM education and the same-industry work experience – to drive strategic outcomes of technological activities. In other words, our results suggest that technology-based firms may lose an advantage in delicately balanced human capital if they lean disproportionately towards general education, STEM education, or work experience in the same-industry when composing the workforce needed for technological development.

The findings of this study carry also practical implications for policy makers and government administrators, particularly with respect to higher educational and industrial policies. The results suggest that policy makers should focus on interventions that enhance STEM education that includes emphasis on practical knowledge and skills. Such policy initiatives may include placing a high priority on improving the quality of the STEM education curriculum and instruction in collaboration with industry and introducing more internship programs. Governments need to consider incentives to encourage students and teachers to be involved in more active domain-specific learning in engineering and science fields. Developing STEM learning programs and providing career advice are likely to also be important to supporting firm’s transition to a higher level of technological intensity.

Our study has limitations. First, the data of this study was collected in Portugal. Future research could extend this study by investigating the role of human capital in other locations or economies that might have different socio-economic contexts. Particularly, future research could adopt comparative perspective and examine how the effects of human capital vary in the different stages of technological catch-up. Second, our study does not track technology development within the same level of technological intensity. Although firms may engage in activities such as incremental improvements or new product creation in contexts often classified as low technology, our dependent variable is measured as a binary indicator and therefore does not capture this diversity. While our approach offers a broad perspective by examining movements across classifications of technological intensity, it may overlook smaller-scale dynamics of technological change that do not result in reclassification. In addition, the measure depends on administrative reclassification in the QP dataset. Although this dataset is rich and well established, it cannot perfectly represent technological progress, since reclassification may sometimes reflect administrative or institutional changes rather than genuine upgrading. For this reason, our analysis should be understood as providing one perspective on technology development rather than a definitive measure. A fuller understanding would be achieved by combining this approach with complementary indicators such as research expenditures, patent counts, or innovation surveys. Future studies could build on this work by directly examining technological advancement within and across categories of technological intensity to provide a more comprehensive view of how human capital influences technology development. Third, human capital management philosophies, policies, and practices may vary among firms and industries, which carries different consequences of technological achievement for firms with the similar level of human capital. For instance, start-ups often have fewer hierarchies and an open culture to facilitate more communication and interaction among workers. Unfortunately, this study was unable to test these issues more carefully because our data does not include information on the human capital management of firms. A more extensive analysis of these issues could shed light on our comprehension of the human capital and technology development link.

## References

[1] Apak S, Atay E. Global Competitiveness in the EU Through Green Innovation Technologies and Knowledge Production. Procedia - Social and Behavioral Sciences. 2015;181:207–17. doi: 10.1016/j.sbspro.2015.04.882

[2] Bjornali ES, Knockaert M, Erikson T. The Impact of Top Management Team Characteristics and Board Service Involvement on Team Effectiveness in High-Tech Start-Ups. Long Range Planning. 2016;49(4):447–63. doi: 10.1016/j.lrp.2015.12.014

[3] Acemoglu D. Introduction to economic growth. Journal of Economic Theory. 2012;147(2):545–50. doi: 10.1016/j.jet.2012.01.023

[4] Acemoglu D, Autor D. What does human capital do? A review of Goldin and Katz’s the race between education and technology. J Econ Lit. 2012;50(2):426–63.

[5] Lee RP, Grewal R. Strategic Responses to New Technologies and Their Impact on Firm Performance. J Mark. 2004;68(4):157–71.

[6] Tsai KH, Wang JC. External technology acquisition and firm performance: A longitudinal study. J Bus Ventur. 2008;23(1):91–112.

[7] Simonen J, McCann P. Firm innovation: The influence of R&D cooperation and the geography of human capital inputs. Journal of Urban Economics. 2008;64(1):146–54. doi: 10.1016/j.jue.2007.10.002

[8] Mu Q, Lee K. Knowledge diffusion, market segmentation and technological catch-up: The case of the telecommunication industry in China. Research Policy. 2005;34(6):759–83.

[9] Faggian A, McCann P. Human capital, graduate migration and innovation in British regions. Cambridge Journal of Economics. 2008;33(2):317–33.

[10] Lee SY, Florida R, Gates G. Innovation, Human Capital, and Creativity. International Review of Public Administration. 2010;14(3):13–24.

[11] Romijn H, Albaladejo M. Determinants of innovation capability in small electronics and software firms in southeast England. Research Policy. 2002;31(7):1053–67. doi: 10.1016/s0048-7333(01)00176-7

[12] Drine I. Institutions, governance and technology catch-up in North Africa. Econ Model. 2012;29(6):2155–62.

[13] Kato M, Okamuro H, Honjo Y. Does Founders’ Human Capital Matter for Innovation? Evidence from Japanese Start-ups. Journal of Small Business Management. 2014;53(1):114–28. doi: 10.1111/jsbm.12094

[14] Alexiev AS, Jansen JJP, Van den Bosch FAJ, Volberda HW. Top Management Team Advice Seeking and Exploratory Innovation: The Moderating Role of TMT Heterogeneity. J Management Studies. 2010;47(7):1343–64. doi: 10.1111/j.1467-6486.2010.00919.x

[15] Talke K, Salomo S, Kock A. Top Management Team Diversity and Strategic Innovation Orientation: The Relationship and Consequences for Innovativeness and Performance. J of Product Innov Manag. 2011;28(6):819–32. doi: 10.1111/j.1540-5885.2011.00851.x

[16] Guo S, Zan B, Sun Y, Zhang M. Effects of top managers’ military experience on technological innovation in the transition economies of China. Technological Forecasting and Social Change. 2020;153:119909. doi: 10.1016/j.techfore.2020.119909

[17] Yang JJ, Li S, Li C. Graduate education and enterprise innovation. Appl Econ Lett. 2025;32(3):391–7.

[18] Barge-Gil A, D’Este P, Herrera L. PhD trained employees and firms’ transitions to upstream R&D activities. Ind Innov. 2021;28(4):424–55.

[19] Hashai N, Zahra S. Founder team prior work experience: An asset or a liability for startup growth? Strategic Entrepreneurship Journal. 2022;16(1):155–84.

[20] McGuirk H, Lenihan H, Hart M. Measuring the impact of innovative human capital on small firms’ propensity to innovate. Research Policy. 2015;44(4):965–76. doi: 10.1016/j.respol.2014.11.008

[21] De Winne S, Sels L. Interrelationships between human capital, HRM and innovation in Belgian start-ups aiming at an innovation strategy. The International Journal of Human Resource Management. 2010;21(11):1863–83. doi: 10.1080/09585192.2010.505088

[22] Solheim MCW, Boschma R, Herstad SJ. Collected worker experiences and the novelty content of innovation. Research Policy. 2020;49(1).

[23] Crown D, Faggian A, Corcoran J. Foreign-Born graduates and innovation: Evidence from an Australian skilled visa program✰,✰✰,★,★★. Research Policy. 2020;49(9):103945. doi: 10.1016/j.respol.2020.103945

[24] Martin-Rojas R, Garcia-Morales VJ, Gonzalez-Alvarez N. Technological antecedents of entrepreneurship and its consequences for organizational performance. Technol Forecast Soc Change. 2019;147:22–35.

[25] Capozza C, Divella M. Human capital and firms’ innovation: evidence from emerging economies. Economics of Innovation and New Technology. 2018;28(7):741–57. doi: 10.1080/10438599.2018.1557426

[26] De Winne S, Sels L. Interrelationships between human capital, HRM and innovation in Belgian start-ups aiming at an innovation strategy. The International Journal of Human Resource Management. 2010;21(11):1863–83. doi: 10.1080/09585192.2010.505088

[27] Hashai N, Zahra SA. A double-edged sword? Founder Teams’ Prior International Experience and INV International Scale-up. Journal of World Business. 2022;57(2):101309. doi: 10.1016/j.jwb.2022.101309

[28] Gimmon E, Levie J. Founder’s human capital, external investment, and the survival of new high-technology ventures. Research Policy. 2010;39(9):1214–26. doi: 10.1016/j.respol.2010.05.017

[29] Fu X, Pietrobelli C, Soete L. The Role of Foreign Technology and Indigenous Innovation in the Emerging Economies: Technological Change and Catching-up. World Development. 2011;39(7):1204–12. doi: 10.1016/j.worlddev.2010.05.009

[30] Honjo Y. The impact of founders’ human capital on initial capital structure: Evidence from Japan. Technovation. 2021;100:102191. doi: 10.1016/j.technovation.2020.102191

[31] Wong PK, Ho YP, Autio E. Entrepreneurship, Innovation and Economic Growth: Evidence from GEM data. Small Business Economics. 2005;24(3):335–50.

[32] Acs ZJ. Innovation and the growth of cities. Urban dynamics and growth: Advances in urban economics. Amsterdam: Elsevier Science & Technology; 2004. p. 635–58.

[33] Wolf M, Terrell D. The high-tech industry, what is it and why it matters to our economic future. 2016.

[34] Cosar AK. Human Capital, Technology Adoption and Development. The BE Journal of Macroeconomics. 2011;11.

[35] Wright M, Hmieleski KM, Siegel DS, Ensley MD. The Role of Human Capital in Technological Entrepreneurship. Entrepreneurship Theory and Practice. 2007;31(6):791–806. doi: 10.1111/j.1540-6520.2007.00202.x

[36] Frantzen D. R&D, human capital and international technology spillovers: a cross-country analysis. Scandinavian Journal of Economics. 2000;102(1):57.

[37] ANI. Portugal remains in the group of moderate innovators. Agência Nacional de Inovação News Release. 2024.

[38] European Commission. Benchmarking national policy frameworks for innovation procurement: Portugal Country Profile. Brussels: DG Research & Innovation; 2024.

[39] WIPO. Global Innovation Index 2024: Portugal Country Profile. World Intellectual Property Organization; 2024.

[40] World Bank G. Portugal human capital country brief. 2024. https://humancapital.worldbank.org/en/country-briefs

[41] Hartmann A. Integriete Organisations-und Technikentwicklung-ein Ansatz zur sach-und bedürrfnisgerechten Gestaltung der Arbeitswelt. Menschengerechte Groupware. 1993. p. 303–28.

[42] Sahoo S, Kumar A, Upadhyay A. How do green knowledge management and green technology innovation impact corporate environmental performance? Understanding the role of green knowledge acquisition. Bus Strategy Environ. 2023;32(1):551–69.

[43] Wang X, Cho SH, Scheller-Wolf A. Green technology development and adoption: competition, regulation, and uncertainty—a global game approach. Management Science. 2020;67(1):201–19. doi: 10.1287/mnsc20193538

[44] Bhattacharya S. Governance and regulation in an emerging technology: Nanotechnology as a case study. 2015. p. 215–8.

[45] OECD. OECD Science, Technology and Industry Scoreboard 2003. 2003.

[46] Eurostat. Eurostat indicators on high-tech industry and knowledge-intensive services. Luxembourg: Eurostat; 2020.

[47] Kato M, Honjo Y. Entrepreneurial human capital and the survival of new firms in high- and low-tech sectors. J Evol Econ. 2015;25(5):925–57.

[48] Colombo MG, Grilli L. Founders’ human capital and the growth of new technology-based firms: A competence-based view. Research Policy. 2005;34(6):795–816. doi: 10.1016/j.respol.2005.03.010

[49] Grilli L, Jensen PH, Murtinu S, Park HD. A close look at the contingencies of founders’ effect on venture performance. Industrial and Corporate Change. 2020;29(4):997–1020. doi: 10.1093/icc/dtaa015

[50] Patzelt H. CEO human capital, top management teams, and the acquisition of venture capital in new technology ventures: An empirical analysis. Journal of Engineering and Technology Management. 2010;27(3–4):131–47. doi: 10.1016/j.jengtecman.2010.06.001

[51] Hsu DH. Experienced entrepreneurial founders, organizational capital, and venture capital funding. Research Policy. 2007;36(5):722–41. doi: 10.1016/j.respol.2007.02.022

[52] Valenti A, Horner S. The human capital of boards of directors and innovation: an empirical examination of the pharmaceutical industry. Int J Innov Mgt. 2019;24(06):2050056. doi: 10.1142/s1363919620500565

[53] Fassio C, Montobbio F, Venturini A. Skilled migration and innovation in European industries. Research Policy. 2019;48(3):706–18. doi: 10.1016/j.respol.2018.11.002

[54] Cao X, Im J. Founder human capital and new technology venture R&D search intensity: the moderating role of an environmental jolt. Small Bus Econ. 2017;50(3):625–42. doi: 10.1007/s11187-017-9911-5

[55] Guo S, Zan B, Sun Y, Zhang M. Effects of top managers’ military experience on technological innovation in the transition economies of China. Technological Forecasting and Social Change. 2020;153:119909. doi: 10.1016/j.techfore.2020.119909

[56] van Uden A, Knoben J, Vermeulen P. Human capital and innovation in Sub-Saharan countries: a firm-level study. Innovation. 2017;19(2):103–24.

[57] De Winne S, Sels L. Interrelationships between human capital, HRM and innovation in Belgian start-ups aiming at an innovation strategy. The International Journal of Human Resource Management. 2010;21(11):1863–83. doi: 10.1080/09585192.2010.505088

[58] Deligianni I, Voudouris I, Spanos Y, Lioukas S. Non-linear effects of technological competence on product innovation in new technology-based firms: Resource orchestration and the role of the entrepreneur’s political competence and prior start-up experience. Technovation. 2019;88:102076. doi: 10.1016/j.technovation.2019.05.002

[59] Vyas V, Vyas R. Human Capital, its Constituents and Entrepreneurial Innovation: A Multi-Level Modeling of Global Entrepreneurship Monitor Data. TIM Review. 2019;9(8):5–17. doi: 10.22215/timreview/1257

[60] Coccia M, Watts J. A theory of the evolution of technology: Technological parasitism and the implications for innovation magement. Journal of Engineering and Technology Management. 2020;55:101552. doi: 10.1016/j.jengtecman.2019.11.003

[61] Papa A, Dezi L, Gregori GL, Mueller J, Miglietta N. Improving innovation performance through knowledge acquisition: the moderating role of employee retention and human resource management practices. JKM. 2018;24(3):589–605. doi: 10.1108/jkm-09-2017-0391

[62] Dakhli M, De Clercq D. Human capital, social capital, and innovation: a multi-country study. Entrepreneurship & Regional Development. 2004;16(2):107–28. doi: 10.1080/08985620410001677835

[63] Nordli AJ. Measuring innovation in tourism with Community Innovation Survey: a first step towards a more valid innovation instruments. Scandinavian Journal of Hospitality and Tourism. 2016;17(4):423–40. doi: 10.1080/15022250.2016.1247382

[64] Chen J, Yin X, Mei L. Holistic innovation: An emerging innovation paradigm. International Journal of Innovation Studies. 2018;2(1):1–13.

[65] Dewangan V, Godse M. Towards a holistic enterprise innovation performance measurement system. Technovation. 2014;34(9):536–45. doi: 10.1016/j.technovation.2014.04.002

[66] You S, Zhou KZ, Jia L. How does human capital foster product innovation? The contingent roles of industry cluster features. J Bus Res. 2021;130.

[67] Markman GD, Siegel DS, Wright M. Research and Technology Commercialization. Journal of Management Studies. 2008;45(8):1401–23.

[68] Zahra SA, Kaul A, Bolívar-Ramos MT. Why Corporate Science Commercialization Fails: Integrating Diverse Perspectives. AMP. 2018;32(1):156–76. doi: 10.5465/amp.2016.0132

[69] Afshari L, Hadian Nasab A. Enhancing organizational learning capability through managing talent: mediation effect of intellectual capital. Human Resource Development International. 2021;24(1):48–64.

[70] Isa ESAI, Muafi M. Human Capital, Organizational Learning and Their Effects on Innovation Behavior and Performance of Banking Employees. IJFBS. 2022;11(1):01–18. doi: 10.20525/ijfbs.v11i1.1533

[71] Sheehan M, Garavan T, Morley M. Training investments and innovation gains in knowledge intensive businesses: The role of firm level human capital and knowledge sharing climate. Human Resource Management Journal. 2025;35(3):667–86.

[72] Schivardi F, Torrini R. Structural change and human capital in the Italian productive system. Giornale degli Economisti. 2010;69(3):119–67.

[73] Singh RK, Agrawal S, Modgil S. Developing human capital 4.0 in emerging economies: an industry 4.0 perspective. Int J Manpow. 2022;43(2):286–309.

[74] Sun T, Abdullah MA. Impact of industrial agglomeration on the upgrading of China’s automobile industry: The threshold effect of human capital and moderating effect of government. Sustainability. 2025;17(7):1–25.

[75] Lenihan H, McGuirk H, Murphy KR. Driving innovation: Public policy and human capital. Research Policy. 2019;48(9):103791. doi: 10.1016/j.respol.2019.04.015

[76] Sulisnaningrum E, Widarni EL, Bawono S. Causality relationship between human capital, technological development and economic growth. Journal of Management, Economics, and Industrial Organization. 2022:1–12.

[77] Danquah M, Amankwah-Amoah J. Assessing the relationships between human capital, innovation and technology adoption: evidence from sub-saharan africa. Technol Forecast Soc Change. 2017;122:24–33.

[78] Pourehtesham M. The relationship between technology and economic growth: The moderating role of human capital. International Journal of Human Capital in Urban Management. 2022;7(4):560–70.

[79] Vandenbussche J, Aghion P, Meghir C. Growth, distance to frontier and composition of human capital. J Econ Growth. 2006;11(2):97–127. doi: 10.1007/s10887-006-9002-y

[80] Bambi PDR, Pea-Assounga JBB. Unraveling the interplay of research investment, educational attainment, human capital development, and economic advancement in technological innovation: A panel VAR approach. Educ Inf Technol. 2025;30(3):3309–41.

[81] Zapata-Cantú L. The Future of Work: Personal and Engaging Practices for a Superior Productivity. Organizational Innovation in the Digital Age. Springer International Publishing. 2022. p. 125–47. doi: 10.1007/978-3-030-98183-9_5

[82] Dakhli M, De Clercq D. Human capital, social capital, and innovation: a multi-country study. Entrepreneurship & Regional Development. 2004;16(2):107–28. doi: 10.1080/08985620410001677835

[83] Romer PM. Endogenous Technological Change. Journal of Political Economy. 1990;98(5, Part 2):S71–102. doi: 10.1086/261725

[84] Fox JT, Smeets V. Does input quality drive measured differences in firm productivity? 2011.

[85] Galindo-Rueda F, Haskel J. Skills, Workforce Characteristics and Firm-Level Productivity: Evidence from the Matched ABI/Employer Skills Survey. 2005.

[86] Lebedinski L, Vandenberghe V. Assessing education’s contribution to productivity using firm-level evidence. Int J Manpow. 2014;35(8):1116–39.

[87] Laursen K, Foss NJ. New human resource management practices, complementarities and the impact on innovation performance. Cambridge J Econ. 2003;27(2):243–63.

[88] Lin CHV, Sanders K. HRM and innovation: a multi‐level organisational learning perspective. Human Resource Management Journal. 2017;27(2):300–17.

[89] Lei H, Khamkhoutlavong M, Le PB. Fostering exploitative and exploratory innovation through HRM practices and knowledge management capability: the moderating effect of knowledge-centered culture. JKM. 2021;25(8):1926–46. doi: 10.1108/jkm-07-2020-0505

[90] Edsand HE. Technological innovation system and the wider context: A framework for developing countries. Technol Soc. 2019;58:101150.

[91] Kaasa A, Vadi M. How does culture contribute to innovation? Evidence from European countries. Economics of Innovation and New Technology. 2010;19(7):583–604. doi: 10.1080/10438590902987222

[92] Zanello G, Fu X, Mohnen P, Ventresca M. The creation and diffusion of innovation in developing countries: A systematic literature review. J Econ Surv. 2016;30(5):884–912.

[93] Becker GS. Front matter, human capital: a theoretical and empirical analysis, with special reference to education. Human Capital: A Theoretical and Empirical Analysis, with Special Reference to Education. Second Edition ed. NBER. 1975. p. 20–2.

[94] Stucki T. How the founders’ general and specific human capital drives export activities of start-ups. Research Policy. 2016;45(5):1014–30. doi: 10.1016/j.respol.2016.02.010

[95] Bilan Y, Mishchuk H, Dzhyhar T. Human capital factors and remuneration: analysis of relations, modelling of influence. Verslas: teorija ir praktika. 2017;18(1):208–14.

[96] Schulz E, Chowdhury S, Van de Voort D. Firm productivity moderated link between human capital and compensation: The significance of task‐specific human capital. Hum Resour Manage. 2013;52(3):423–39.

[97] Domurath A, Patzelt H. Founder non-international experience and venture internationalization. J Int Entrep. 2019;17(4):494–519. doi: 10.1007/s10843-019-00249-0

[98] Sianesi B, Reenen JV. The Returns to Education: Macroeconomics. Journal of Economic Surveys. 2003;17(2):157–200. doi: 10.1111/1467-6419.00192

[99] Krueger AB, Lindahl M. Education for Growth: Why and for Whom? Journal of Economic Literature. 2001;39(4):1101–36. doi: 10.1257/jel.39.4.1101

[100] Lebedinski L, Vandenberghe V. Assessing education’s contribution to productivity using firm-level evidence. Vincent V, editor. Int J Manpow. 2014;35(8):1116–39.

[101] Hatch NW, Dyer JH. Human capital and learning as a source of sustainable competitive advantage. Strategic Management Journal. 2004;25(12):1155–78. doi: 10.1002/smj.421

[102] Rauch A, Frese M, Utsch A. Effects of human capital and long–term human resources development and utilization on employment growth of small–scale businesses: A causal analysis. Entrepreneurship Theory and Practice. 2005;29(6):681–98.

[103] Smith KG, Collins CJ, Clark KD. Existing Knowledge, Knowledge Creation Capability, and the Rate of New Product Introduction in High-Technology Firms. AMJ. 2005;48(2):346–57. doi: 10.5465/amj.2005.16928421

[104] Madsen JB. Human capital and the world technology frontier. Review of Economics and Statistics. 2014;96(4).

[105] Kämpfen F, Maurer J. Does education help “old dogs” learn “new tricks”? The lasting impact of early-life education on technology use among older adults. Research Policy. 2018;47(6):1125–32. doi: 10.1016/j.respol.2018.03.017

[106] Marvel MR, Lumpkin GT. Technology entrepreneurs’ human capital and its effects on innovation radicalness. Entrepreneurship Theory and Practice. 2007;31(6):807–28.

[107] de los Dolores González M, Husted BW. Gender, human capital, and opportunity identification in Mexico. International Journal of Gender and Entrepreneurship. 2011;3(3):236–53. doi: 10.1108/17566261111169322

[108] Schneider L, Günther J, Brandenburg B. Innovation and skills from a sectoral perspective: a linked employer–employee analysis. Economics of Innovation and New Technology. 2010;19(2):185–202. doi: 10.1080/10438590902872887

[109] Crook TR, Todd SY, Combs JG, Woehr DJ, Ketchen DJJ. Does human capital matter? A meta-analysis of the relationship between human capital and firm performance. Journal of Applied Psychology. 2011;96(3):443–56. 21244126 10.1037/a0022147

[110] Lafuente E, Rabetino R. Human capital and growth in Romanian small firms. Journal of Small Business and Enterprise Development. 2011;18(1):74–96. doi: 10.1108/14626001111106442

[111] Lee Y-N, Walsh JP. Inventing while you work: Knowledge, non-R&D learning and innovation. Research Policy. 2016;45(1):345–59. doi: 10.1016/j.respol.2015.09.009

[112] Robson PJA, Akuetteh CK, Westhead P, Wright M. Innovative opportunity pursuit, human capital and business ownership experience in an emerging region: evidence from Ghana. Small Bus Econ. 2011;39(3):603–25. doi: 10.1007/s11187-011-9333-8

[113] Ganotakis P. Founders’ human capital and the performance of UK new technology based firms. Small Bus Econ. 2010;39(2):495–515. doi: 10.1007/s11187-010-9309-0

[114] Oakey RP. Technical entreprenenurship in high technology small firms: some observations on the implications for management. Technovation. 2003;23(8):679–88. doi: 10.1016/s0166-4972(03)00045-2

[115] Ravasi D, Turati C. Exploring entrepreneurial learning: a comparative study of technology development projects. J Bus Ventur. 2005;20(1):137–64.

[116] Madsen H, Neergaard H, Ulhøi JP. Knowledge‐intensive entrepreneurship and human capital. Jrnl of Small Bus Ente Dev. 2003;10(4):426–34. doi: 10.1108/14626000310504738

[117] Colombo MG, Delmastro M, Grilli L. Entrepreneurs’ human capital and the start-up size of new technology-based firms. Int J Ind Organ. 2004;22(8):1183–211.

[118] Gimeno J, Folta TB, Cooper AC, Woo CY. Survival of the Fittest? Entrepreneurial Human Capital and the Persistence of Underperforming Firms. Administrative Science Quarterly. 1997;42(4):750–83.

[119] Bianchi N, Giorcelli M. Scientific education and innovation: from technical diplomas to university stem degrees. J Eur Econ Assoc. 2020;18(5):2608–46.

[120] Cohen WM, Levinthal DA. Absorptive Capacity: A New Perspective on Learning and Innovation. Adm Sci Q. 1990;35(1):128–52.

[121] Jiménez-Barrionuevo MM, García-Morales VJ, Molina LM. Validation of an instrument to measure absorptive capacity. Technovation. 2011;31(5–6):190–202. doi: 10.1016/j.technovation.2010.12.002

[122] Colombo MG, Delmastro M. How effective are technology incubators? Research Policy. 2002;31(7):1103–22. doi: 10.1016/s0048-7333(01)00178-0

[123] Hobday M. Innovation in Asian Industrialization: A Gerschenkronian Perspective. Oxford Development Studies. 2003;31(3):293–314. doi: 10.1080/1360081032000111715

[124] Agénor PR, Dinh HT. From imitation to innovation: Public policy for industrial transformation. Washington, DC: World Bank; 2013.

[125] Bell M, Figueiredo PN. Innovation capability building and learning mechanisms in latecomer firms: recent empirical contributions and implications for research. Canadian Journal of Development Studies/Revue canadienne d’études du développement. 2012;33(1):14–40. doi: 10.1080/02255189.2012.677168

[126] Shane S. Prior Knowledge and the Discovery of Entrepreneurial Opportunities. Organization Science. 2000;11(4):448–69. doi: 10.1287/orsc.11.4.448.14602

[127] Lin Y, Rasiah R. Human capital flows in Taiwan’s technological catch up in integrated circuit manufacturing. J Contemp Asia. 2014;44(1):64–83.

[128] Kwark NS, Shyn YS. International R&D spillovers revisited: Human capital as an absorptive capacity for foreign technology. Int Econ J. 2006;20(2).

[129] Enders J. Research training and careers in transition: a European perspective on the many faces of the Ph.D. Studies in Continuing Education. 2004;26(3):419–29.

[130] Hancock S, Walsh E. Beyond knowledge and skills: rethinking the development of professional identity during the STEM doctorate. Studies in Higher Education. 2014;41(1):37–50. doi: 10.1080/03075079.2014.915301

[131] De Grande H, De Boyser K, Vandevelde K, Van Rossem R. From Academia to Industry: Are Doctorate Holders Ready? J Knowl Econ. 2014;5(3):538–61. doi: 10.1007/s13132-014-0192-9

[132] Borah D, Malik K, Massini S. Are engineering graduates ready for R&D jobs in emerging countries? Teaching-focused industry-academia collaboration strategies. Research Policy. 2019;48(9):103837. doi: 10.1016/j.respol.2019.103837

[133] Hayter CS, Parker MA. Factors that influence the transition of university postdocs to non-academic scientific careers: An exploratory study. Research Policy. 2019;48(3):556–70. doi: 10.1016/j.respol.2018.09.009

[134] Salminen-Karlsson M, Wallgren L. The interaction of academic and industrial supervisors in graduate education. High Educ. 2008;56(1):77–93.

[135] Stenard BS, Sauermann H. Educational Mismatch, Work Outcomes, and Entry Into Entrepreneurship. Organization Science. 2016;27(4):801–24. doi: 10.1287/orsc.2016.1071

[136] Buenstorf G, Heinisch DP. When do firms get ideas from hiring PhDs? Research Policy. 2020;49(3):103913. doi: 10.1016/j.respol.2019.103913

[137] Box S. OECD work on innovation – a stocktaking of existing work. Paris. 2009.

[138] Kim W, Shi Y, Gregory M. Transition from imitation to innovation: lessons from a Korean Multinational Corporation.(Report). International Journal of Business. 2004;9(4):329.

[139] Lee K. Making a Technological Catch‐up: Barriers and opportunities. Asian Journal of Technology Innovation. 2005;13(2):97–131. doi: 10.1080/19761597.2005.9668610

[140] Instituto Nacional de Estatística. Empresas em Portugal: 2019. Lisbon. 2021.

[141] Nesta L, Saviotti PP. Coherence of the knowledge base and the firm’s innovative performance: evidence from the US pharmaceutical industry. J Ind Econ. 2005;53(1):123–42.

[142] Zoghi C, Mohr RD, Meyer PB. Workplace organization and innovation. Canadian J of Economics. 2010;43(2):622–39. doi: 10.1111/j.1540-5982.2010.01586.x

[143] Zhou H, Dekker R, Kleinknecht A. Flexible labor and innovation performance: evidence from longitudinal firm-level data. Industrial and Corporate Change. 2011;20(3):941–68. doi: 10.1093/icc/dtr013

[144] Henriques MH, Pena dos Reis R, Garcia GG, João P, Marques RM, Custódio S. Developing paleogeographic heritage concepts and ideas through the Upper Jurassic record of the Salgado and Consolação geosites (Lusitanian Basin, Portugal). Resources Policy. 2022;76:102594. doi: 10.1016/j.resourpol.2022.102594

[145] Conselho Nacional da Educação. O estado da educação 2020 [The state of education 2020]. Lisboa; 2021.

[146] Bongers A, Díaz-Roldán C, Torres JL. Highly Skilled International Migration, STEM Workers, and Innovation. Economics. 2022;16(1):73–89.

[147] Szczepanska-Woszczyna K, Lyulyov O, Pimonenko T, Infante-Moro A, Zimbroff A, Solodovnikov S. The Contribution of Higher Education to Innovation Development: Long-Term and Short-Term Analysis. Forum Scientiae Oeconomia. 2024;12(4):8–29.

[148] Stewart F. STEM and the local economy: Do regions reap the benefits of a STEM-educated workforce? Employment Research Newsletter. 2018;25(1):1.

[149] Wang AX, Zhou KZ. Financial munificence, R&D intensity, and new venture survival: critical roles of CEO attributes. Small Bus Econ. 2022;59(4):1641–59. doi: 10.1007/s11187-021-00592-4

[150] Teixeira AAC, Tavares-Lehmann AT. Human capital intensity in technology-based firms located in Portugal: Does foreign ownership matter? Research Policy. 2014;43(4):737–48. doi: 10.1016/j.respol.2014.01.001

[151] Lorentzen J, Møllgaard P, Rojec M. Host-country absorption of technology: Evidence from automotive supply networks in Eastern Europe. Ind Innov. 2003;10(4):415–32.

[152] Honjo Y, Kato M. Do initial financial conditions determine the exit routes of start-up firms? J Evol Econ. 2019;29(3):1119–47. doi: 10.1007/s00191-019-00623-0

[153] Agarwal R, Audretsch DB. Does Entry Size Matter? The Impact of the Life Cycle and Technology on Firm Survival. The J Industrial Economics. 2001;49(1):21–43. doi: 10.1111/1467-6451.00136

[154] Yi J, Hong J, Hsu W, Wang C. The role of state ownership and institutions in the innovation performance of emerging market enterprises: Evidence from China. Technovation. 2017;62–63:4–13. doi: 10.1016/j.technovation.2017.04.002

[155] Chakrabarti AK, Halperin MR. Technical performance and firm size: Analysis of patents and publications of U.S. firms. Small Bus Econ. 1990;2(3):183–90. doi: 10.1007/bf00389526

[156] Stock GN, Greis NP, Fischer WA. Absorptive capacity and new product development. The Journal of High Technology Management Research. 2001;12(1):77–91. doi: 10.1016/s1047-8310(00)00040-7

[157] Lenaerts K, Merlevede B. Firm size and spillover effects from foreign direct investment: the case of Romania. Small Bus Econ. 2015;45(3):595–611. doi: 10.1007/s11187-015-9652-2

[158] The Commission of the European Community. COMMISSION RECOMMENDATION of 6 May 2003 concerning the definition of micro, small and medium-sized enterprises. Official Journal of the European Union; 2003;46:36.

[159] Testa G, Szkuta K. Improving access to finance for young innovative enterprises with growth potential: evidence of impact on firms’ output Part 2. R&D grant schemes: lessons learned from evaluations. Luxembourg: Publications Office of the European Union; 2018.

[160] García-Quevedo J, Pellegrino G, Vivarelli M. R&D drivers and age: Are young firms different? Research Policy. 2014;43(9):1544–56. doi: 10.1016/j.respol.2014.04.003

[161] Norton EC, Dowd BE, Maciejewski ML. Marginal Effects-Quantifying the Effect of Changes in Risk Factors in Logistic Regression Models. JAMA. 2019;321(13):1304–5. doi: 10.1001/jama.2019.1954 30848814

[162] Kashyap AS, Swastik S. Regression model to predict bike sharing demand. Int J Innov Sci Res Technol. 2021;6(3):1024–8.

[163] Wooldridge JM. Introductory econometrics: A modern approach. Boston: Cengage Learning; 2015.

[164] King G, Zeng L. Logistic Regression in Rare Events Data. Polit anal. 2001;9(2):137–63. doi: 10.1093/oxfordjournals.pan.a004868

[165] Ernst & Young. Porto and Northern Portugal: A Magnet for Investment. 2019.

[166] Ernst & Young. How can a resilient Portugal become a platform for sustainable investment in the future?. 2020.

[167] Instituto Nacional de Estatística. Região Norte em números: 2019. Lisboa: Instituto Nacional de Estatística; 2021.

[168] Instituto Nacional de Estatística. Area Metropolitana de Lisboa em Números: 2019. Lisboa: Instituto Nacional de Estatística; 2021.

[169] Instituto Nacional de Estatística. Região Algarve em números: 2019. Lisboa: Instituto Nacional de Estatística; 2021.

[170] Chun D, Chung Y, Bang S. Impact of firm size and industry type on R&D efficiency throughout innovation and commercialisation stages: evidence from Korean manufacturing firms. Technol Anal Strateg Manag. 2015;27(8):895–909.

[171] Fang X, Paez NR, Zeng B. The nonlinear effects of firm size on innovation: an empirical investigation. Economics of Innovation and New Technology. 2021;30(1):48–65.

[172] Li Y, Zhang YA, Shi W. Navigating geographic and cultural distances in international expansion: The paradoxical roles of firm size, age, and ownership. Strategic Management Journal. 2019;41(5):921–49. doi: 10.1002/smj.3098

[173] Coad A, Segarra A, Teruel M. Innovation and firm growth: Does firm age play a role? Research Policy. 2016;45(2):387–400. doi: 10.1016/j.respol.2015.10.015

[174] Coad A, Daunfeldt S-O, Halvarsson D. Bursting into life: firm growth and growth persistence by age. Small Bus Econ. 2017;50(1):55–75. doi: 10.1007/s11187-017-9872-8

[175] Wöhrl R, Hüsig S, Dowling M. The interaction of R&D intensity and firm age: Empirical evidence from technology-based growth companies in the German “Neuer Markt”. The Journal of High Technology Management Research. 2009;20(1):19–30. doi: 10.1016/j.hitech.2009.02.006

[176] Firth D. Bias reduction of maximum likelihood estimates. Biometrika. 1993;80(1):27–38.

[177] Thies F, Huber A, Bock C, Benlian A, Kraus S. Following the crowd—does crowdfunding affect venture capitalists’ selection of entrepreneurial ventures? Journal of Small Business Management. 2019;57(4):1378–98.

[178] Laplume AO, Xavier-Oliveira E, Dass P, Thakur R. The organizational advantage in early inventing and patenting: Empirical evidence from interference proceedings. Technovation. 2015;43–44:40–8. doi: 10.1016/j.technovation.2015.03.005

[179] Archibugi D, Denni M, Filippetti A. The technological capabilities of nations: The state of the art of synthetic indicators. Technological Forecasting and Social Change. 2009;76(7):917–31. doi: 10.1016/j.techfore.2009.01.002

[180] Smith KG, Collins CJ, Clark KD. Existing Knowledge, Knowledge Creation Capability, and the Rate of New Product Introduction in High-Technology Firms. AMJ. 2005;48(2):346–57. doi: 10.5465/amj.2005.16928421

[181] Madsen JB. Human capital and the world technology frontier. Rev Econ Stat. 2014;96(4):676–92.

[182] Shane S. Prior Knowledge and the Discovery of Entrepreneurial Opportunities. Organization Science. 2000;11(4):448–69. doi: 10.1287/orsc.11.4.448.14602

[183] De Grande H, De Boyser K, Vandevelde K, Van Rossem R. From Academia to Industry: Are Doctorate Holders Ready? J Knowl Econ. 2014;5(3):538–61. doi: 10.1007/s13132-014-0192-9

[184] Salminen-Karlsson M, Wallgren L. The interaction of academic and industrial supervisors in graduate education: an investigation of industrial research schools. High Educ. 2008;56(1):77–93.

[185] Stenard BS, Sauermann H. Educational Mismatch, Work Outcomes, and Entry Into Entrepreneurship. Organization Science. 2016;27(4):801–24. doi: 10.1287/orsc.2016.1071

[186] Buenstorf G, Heinisch DP. When do firms get ideas from hiring PhDs? Res Policy. 2020;49(3):103913.

[187] Hancock S, Walsh E. Beyond knowledge and skills: rethinking the development of professional identity during the STEM doctorate. Studies in Higher Education. 2014;41(1):37–50. doi: 10.1080/03075079.2014.915301

[188] Hayter CS, Parker MA. Factors that influence the transition of university postdocs to non-academic scientific careers: An exploratory study. Research Policy. 2019;48(3):556–70. doi: 10.1016/j.respol.2018.09.009

[189] Box S. OECD Work on Innovation – A Stocktaking of Existing Work. OECD Science, Technology and Industry Working Papers. 2009 Feb 2;2009/02.

[190] Borah D, Malik K, Massini S. Are engineering graduates ready for R&D jobs in emerging countries? Teaching-focused industry-academia collaboration strategies. Research Policy. 2019;48(9).

[191] Kim W, Shi Y, Gregory M. Transition from imitation to innovation: lessons learnt from a Korean multinational corporation. International Journal of Business. 2011;9.

[192] Lee K. Making a Technological Catch‐up: Barriers and opportunities. Asian Journal of Technology Innovation. 2005;13(2):97–131. doi: 10.1080/19761597.2005.9668610

